# Synchronous primary gastric and renal tumors: a case report

**DOI:** 10.2144/fsoa-2023-0009

**Published:** 2023-05-16

**Authors:** Salma Merhaben, Asma Ben Mohamed, Amal Khsiba, Moufida Mahmoudi, Salwa Nechi, Emna Chelbi, Houda Kilani, Manel Yakoubi, Mohamed Lamine Hamzaoui, Mouna Medhioub, Mohamed Moussadak Azouz

**Affiliations:** 1 Gastroenterology Department, Mohamed Taher Maamouri Hospital, Eezzdine Chelbi Avenue Mrezgua, Nabeul, 8000, Tunisia; 2 Histopathology Department, Mohamed Taher Maamouri Hospital,Eezzdine Chelbi Avenue Mrezgua, Nabeul, 8000, Tunisia

**Keywords:** clear cell renal carcinoma, gastric cancer, multiple primary malignancies, renal cancer, signet ring cells

## Abstract

Synchronous multiple primary cancers of the stomach and kidney are very rare, only 45 cases of synchronous multiple primary cancers of the stomach and kidney had been reported in the literature up until 2020. Thus far, no particular risk factors have been identified. We present a case of synchronous multiple primary cancers of the stomach and kidney in a 67-year-old female presenting with a 3-month history of vomiting and abdominal pain. The diagnosis of gastric adenocarcinoma with signet ring cells was confirmed through upper endoscopy with biopsies, while CT-guided biopsies of the renal tumor confirmed the diagnosis of primary kidney neoplasm.

## Plain language summary

Having more than one cancer at the same time is known as multiple primary malignancies. Having cancers in both the stomach and kidney at the same time is even rarer, with only 45 cases reported in literature. The exact causes of such cancers occurring together are not yet known. We present a 67-year-old woman who was diagnosed with synchronous multiple primary cancers of the stomach and kidney. She presented with vomiting and abdominal pain. The diagnosis of gastric cancer was confirmed through upper endoscopy with biopsies, while biopsies of the renal tumor confirmed the diagnosis of primary kidney cancer.

The number of cases of multiple primary malignancies has risen in recent years, this is partly attributed to the aging population and advancements in diagnostic techniques. The prevalence of multiple primary neoplasms ranges from 0.73 to 11.7% [1,2].

Warren and Gates proposed three diagnostic criteria for multiple primary malignancies in 1932: each tumor must present definite features of malignancy; each neoplasm must be topographically separate and distinct; and the possibility of one being a metastasis of the other must be ruled out [3]. Synchronous tumors refer to cases in which a second primary cancer is diagnosed within 6 months of the initial neoplasm [3].

Gastric cancer is a common diagnosis, with over one million cases reported worldwide each year. It is the fifth-most commonly diagnosed cancer globally [2]. The incidence of another synchronous primary tumor being present along with a primary gastric cancer varies from 0.7 to 11% [4–8]. with the most common synchronous sites being colorectal, lung and liver cancer [8,9].

The simultaneous diagnosis of primary renal and gastric malignancies is even rarer. Only 45 cases of synchronous multiple primary cancers of the stomach and kidney had been reported in the literature up until 2020.

We have an intriguing case study to present, featuring a 67-year-old female patient with synchronous renal cell carcinoma and gastric adenocarcinoma.

## Case report

A 67-year-old female patient presented with a 3-month history of vomiting and abdominal pain. She reported poor appetite and a 10 kg weight loss in the last 3 months.

She did not have any significant family history, but she had a personal history of diabetes mellitus Type 2 and hypertension. Her diabetes was well controlled with insulin, and her arterial hypertension was stable with the use of calcium channel blockers.

She did not report any cigarette or alcohol consumption.

The patient weighed 47 kg and had a low BMI of 17.3 kg/m^2^. Her physical examination revealed anicteric sclera and dehydration signs with dry mucous membranes. Her blood pressure was normal, but her heart rate was elevated at 110 beats per min. Epigastric tenderness was noted. No peripheral lymph nodes or organomegaly were observed. Digital rectal examination revealed no abnormalities.

Routine hematological and biochemical investigations revealed a low hemoglobin level of 8 g/dl (normal range: 12.00–16.00 g/dl), normal platelet level of 354,000/mm^3^, a normal CRP level, severe hypokalemia of 1.9 mmol/l and acute renal failure with serum urea of 20 mmol/l (normal range: 4–8 mmol/l) and serum creatinine of 186 µmol/l (normal range: 62–106 µmol/l).

The other laboratory data were as follows: total bilirubin was 7 mg/l, AST was 30 IU/l; ALT was 25 IU/l; ALP was 100 U/ml and GGT was 32UI/l.

CEA and CA19-9 levels were normal.

The following tests were also performed and were all negative: hepatitis C virus antibody, hepatitis B virus antigen and HIV antibody.

**Figure 1.**
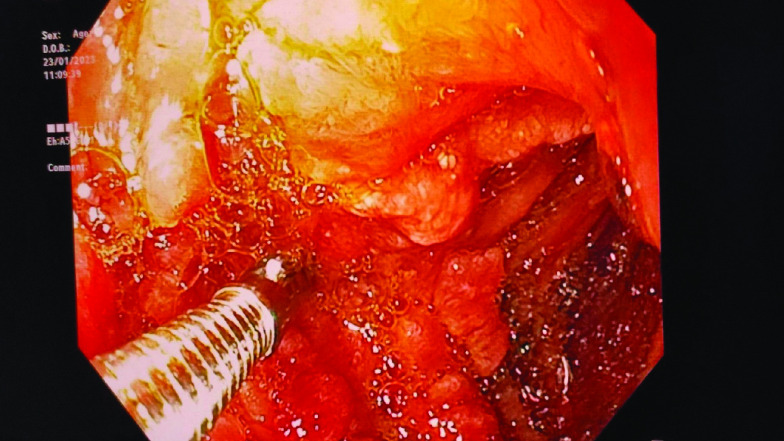
Upper endoscopy showing the antropyloric tumor.

The first diagnostic procedure to perform in a patient presenting with vomiting and abdominal pain is upper endoscopy. It revealed large ulcerated folds in the antropyloric region, which narrowed the lumen (Figure 1).

**Figure 2.**
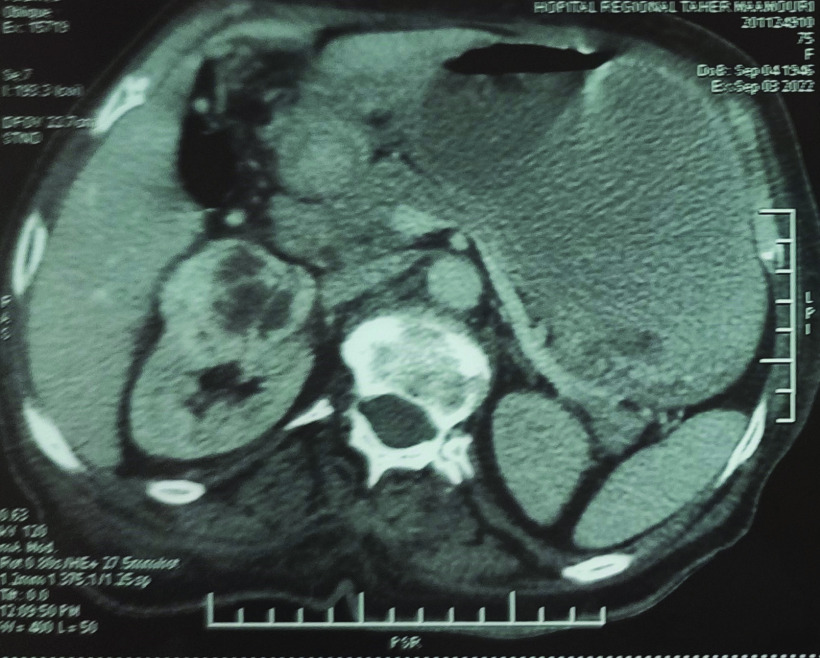
A computed tomography image showing an exophytic hypervascular mass of the inferior pole of the left kidney.

**Figure 3.**
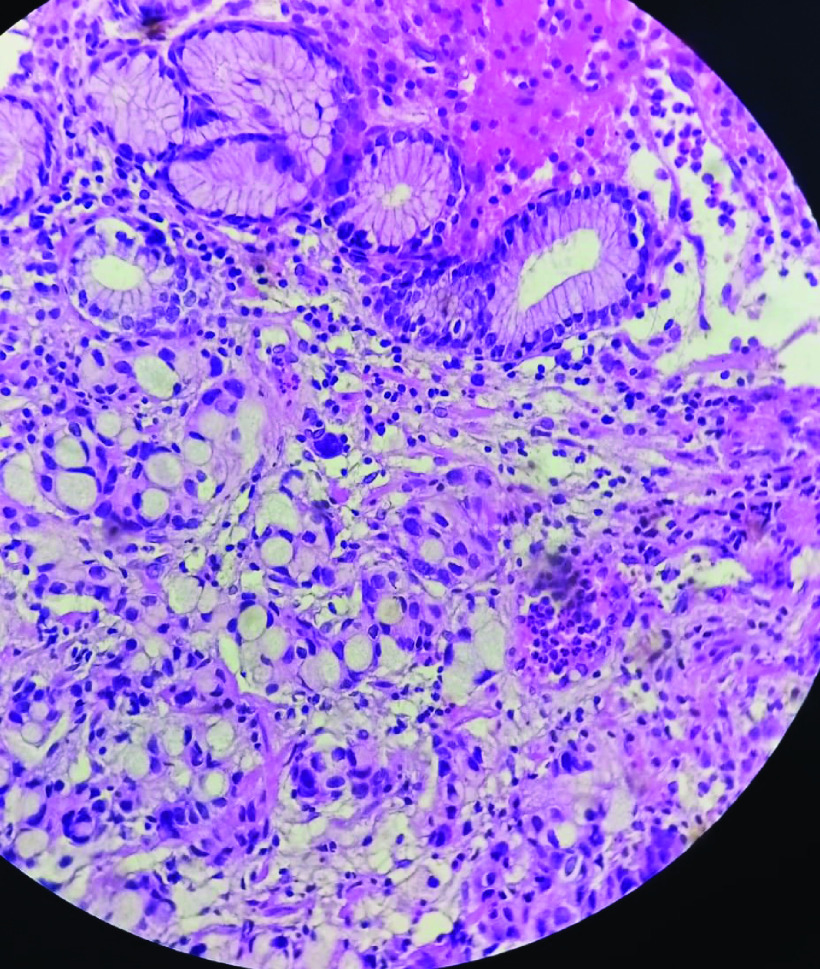
Histopathological examination of antrum and pylorus biopsies with hematoxylin and eosin (H&E ×200) showing the gastric adenocarcinoma with signet ring cells.

**Figure 4.**
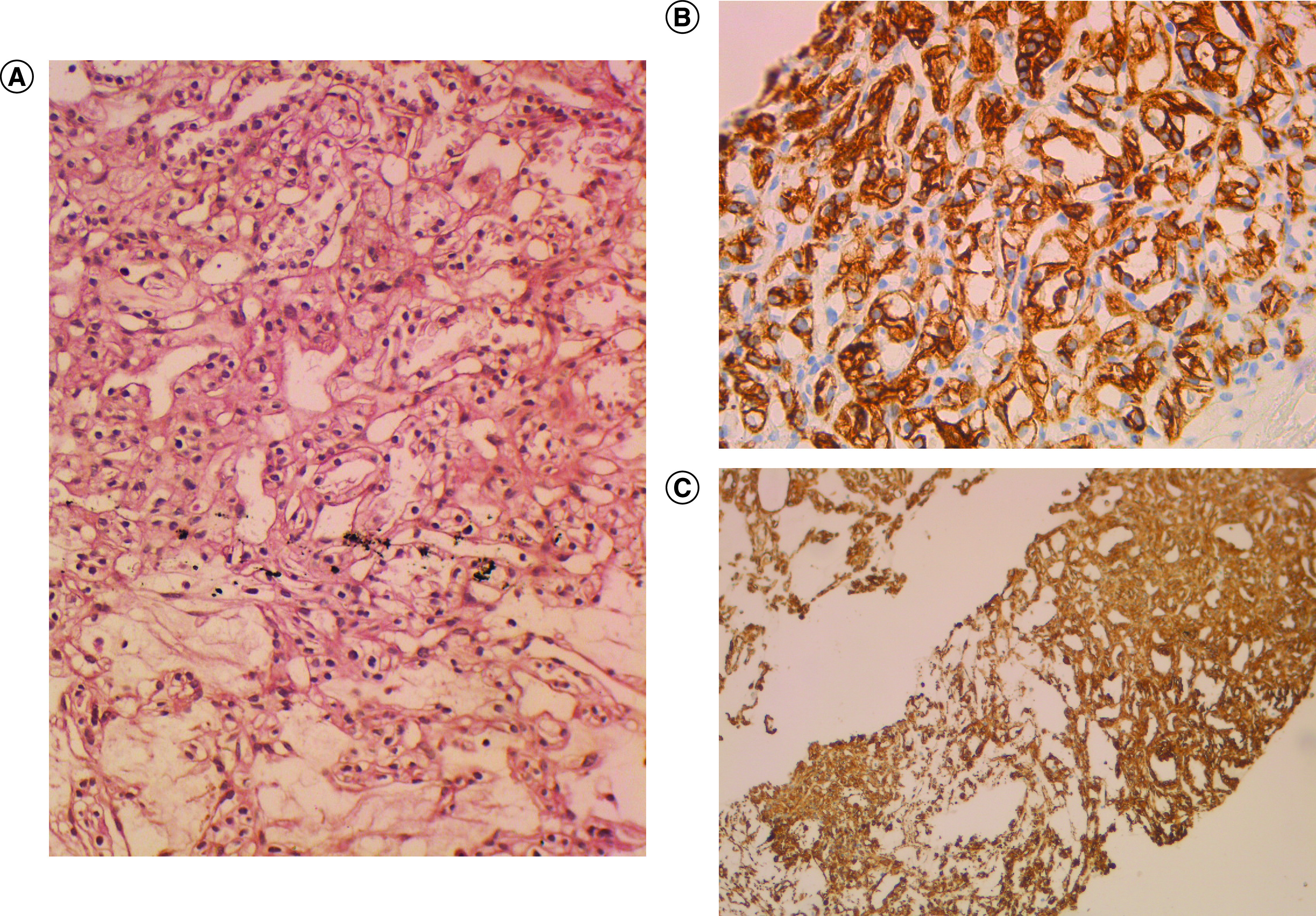
Histopathological and immunohistochemical features of kidney biopsy. **(A)** Renal carcinomatous proliferation with tubular and alveolar architecture and clear tumor cells in microscopic examination of kidney biopsy with hematoxylin and eosin (H&E ×200). **(B)** Clear renal tumor cells that express CK7 (immunohistochemistry [IHC] ×200). **(C)** Clear renal tumor cells that express vimentin (IHC ×200).

The histological examination of gastric tissue samples revealed a gastric adenocarcinoma with signet ring cells (Figure 2). A thoracic abdominal pelvic CT scan revealed an irregular thickening of the antropyloric region, as well as a 7-mm pulmonary nodule. The scan also identified two renal masses, one on the right and one on the left, measuring 55 × 40 mm and 10 × 15 mm, respectively (Figure 3). No liver metastases or peritoneal carcinomatosis were detected. The left renal tumor was biopsied using computed tomography guidance. The histological examination and immunohistochemistry confirmed The diagnosis of a primary kidney neoplasm by revealing a proliferation of carcinoma cells with clear renal cells that expressed CK7 and vimentin. (Figures 4A– C).

A diagnostic laparoscopy was conducted, which revealed a peritoneal effusion. No peritoneal nodules were found. The histopathological examination of a biopsy from the omentum confirmed the presence of neoplastic invasion.

Considering the severe prognosis, there was no discernible advantage in investigating the pulmonary nodule.

The patient was referred to the oncology department for palliative chemotherapy.

## Discussion

In our case, we observed two different types of malignant tumors in a female patient: adenocarcinoma of the stomach and clear cell renal carcinoma. Upon reviewing the literature, we found that synchronous renal cell neoplasms are extremely rare, with a prevalence of 0.11–0.37% [8]. Additionally, the male-to-female ratio is 2:1 and most renal tumors are discovered incidentally through imaging studies [8].

In fact, as of 2020, there have only been 45 reported cases of synchronous multiple primary cancers of the stomach and kidney in the literature [8].

Chronic smoking, advanced age (over 60 years) and the male sex have been identified as risk factors for synchronous gastric and renal tumors in the review of Martín-Pérez *et al.* [8].

Another observation made was that there is no identified genetic alteration or association that links the synchronous presentation of gastric and renal cancers [8].

The common clinical manifestations observed were gastrointestinal bleeding, abdominal pain, unexplained weight loss and the absence of upper or lower urinary tract symptoms, which was not the case with our patient as she did not exhibit gastrointestinal bleeding, but instead presented with vomiting as the main symptom [8].

In the literature, poorly differentiated adenocarcinoma is reported as the most common histopathological type of gastric cancer [8]. Similarly, cell carcinoma is documented as the most common histopathological type of renal carcinoma. This aligns with the histopathological findings in our patient’s case [8].

For the treatment, and according to a report by Yoshino *et al.*, the prognosis of patients with multiple primary cancers is determined independently by the stage of each neoplasm [10]. The preferred surgical treatment for multiple primary cancers is curative resection of each cancer [10]. However, as these cancers are more commonly found in elderly patients, it is important to carefully consider the potential for a decline in quality of life when considering radical resection [11]. In Martín-Pérez *et al.*’s review, the most commonly reported treatment was simultaneous resection of both primary tumors, typically using partial gastrectomy with anastomosis and radical nephrectomy [8]. However, our patient’s case was different as the gastric tumor was at an advanced stage with peritoneal carcinomatosis.

## Conclusion

To summarize, we present a unique case of synchronous double primary malignancies involving the stomach and kidney in a 67-year-old woman. The diagnosis of gastric adenocarcinoma with signet ring cells was confirmed through upper endoscopy with biopsies, while CT-guided biopsies of the renal tumor confirmed the diagnosis of primary kidney neoplasm. In addition to the rarity of the subject, our case has two specificities: The first is the female gender since most of the case reports concern male patients and the second is the bilateral renal involvement.

It is important to consider the possibility of synchronous multiple primary malignancies, especially in elderly patients and to note that the presence of multiple primary neoplasms does not necessarily indicate a poor prognosis.

**Executive summary**

- In 1932, Warren and Gates introduced three diagnostic criteria for multiple primary malignancies.
- The occurrence of primary malignancies in both the kidney and stomach at the same time is rare, with only 45 reported cases in the literature up until 2020.
- There is a lack of literature on the subject.
- Risk factors for synchronous tumors in the stomach and kidney include chronic smoking, advanced age (over 60 years) and male gender.
- No specific risk factors have been identified.
- We report a case of a 67-year-old woman with synchronous double primary malignancies in the stomach and kidney who presented with vomiting and abdominal pain for 3 months. The diagnosis of gastric adenocarcinoma with signet ring cells was confirmed by upper endoscopy with biopsies, while CT-guided biopsies confirmed the diagnosis of primary kidney neoplasm.
- Diagnostic laparoscopy was performed, and carcinomatosis was confirmed.
- It is crucial to consider the possibility of synchronous multiple primary malignancies, especially in elderly patients.
- The presence of multiple primary neoplasms does not necessarily indicate a poor prognosis.
